# Enzymatically active apurinic/apyrimidinic endodeoxyribonuclease 1 is released by mammalian cells through exosomes

**DOI:** 10.1016/j.jbc.2021.100569

**Published:** 2021-03-19

**Authors:** Giovanna Mangiapane, Isabella Parolini, Kristel Conte, Matilde Clarissa Malfatti, Jessica Corsi, Massimo Sanchez, Agostina Pietrantoni, Vito G. D’Agostino, Gianluca Tell

**Affiliations:** 1 Laboratory of Molecular Biology and DNA repair, Department of Medicine (DAME), University of Udine, Udine, Italy; 2 Department of Oncology and Molecular Medicine, Istituto Superiore di Sanità, Rome, Italy; 3 Department of Cellular, Computational and Integrative Biology (CIBIO), University of Trento, Trento, Italy; 4 Core Facilities, Istituto Superiore di Sanità, Rome, Italy

**Keywords:** apurinic/apyrimidinic endodeoxyribonuclease 1, base excision repair biomarker, extracellular vesicles, exosomes, genotoxic damage, proteasome, alpha, amplified luminescence proximity homogeneous assay, AML, acute myeloid leukemia, APE1/Ref1, apurinic/apyrimidinic endodeoxyribonuclease 1/redox factor1, BER, base excision repair, CDDP, cisplatin, CM, conditioned medium, DAMP, damage-associated molecular pattern, DDR, DNA damage response, Doxo, doxorubicin, ds_dF:dC, abasic deoxyribonucleotides in dsDNA, ds_r8oxoG:dC, oxidized ribonucleotide embedded in dsDNA, ds_rF:dC, abasic ribonucleotide embedded in dsDNA, DSB, double-strand breaks, ESCRT, endosomal sorting complex required for transport, EVs, extracellular vesicles, EVs/exo, EVs containing exosomes, EXE, EVs extract, GM130, Golgi apparatus protein, HMDS, hexamethyldisilazane, hnRNP A2/B1, heterogeneous nuclear ribonucleoprotein A2/B1, HCC, hepatocellular carcinoma, ILVs, intraluminal vesicles, miRNA, microRNA, MVBs, multivesicular bodies, NBI, nickel-based isolation, NCE, nuclear cell extracts, ncRNA, noncoding RNA, NPM1, nucleophosmin 1, NSCLC, nonsmall-cell lung cancer, NTA, nanoparticle tracking analysis, RAGE, receptor for advanced glycation end product, rAPE1^WT^, recombinant APE1^WT^ protein, rGST-APE1^K4pleA^, recombinant GST APE1^K4pleA^ protein, rGST-APE1^NΔ33^, recombinant GST APE1^NΔ33^ protein, rGST-APE1^WT^, recombinant GST APE1^WT^ protein, rGST, recombinant GST, sAPE1, serum APE1, SASP, senescence-associated secretory phenotype, SEM, scanning electron microscopy, TNBC, triple-negative breast cancer, TRPS, tunable resistive pulse sensing, TSA, trichostatin A, WCE, whole-cell extract

## Abstract

The apurinic/apyrimidinic endodeoxyribonuclease 1 (APE1), the main AP-endonuclease of the DNA base excision repair pathway, is a key molecule of interest to researchers due to its unsuspected roles in different nonrepair activities, such as: i) adaptive cell response to genotoxic stress, ii) regulation of gene expression, and iii) processing of microRNAs, which make it an excellent drug target for cancer treatment. We and others recently demonstrated that APE1 can be secreted in the extracellular environment and that serum APE1 may represent a novel prognostic biomarker in hepatocellular and non-small-cell lung cancers. However, the mechanism by which APE1 is released extracellularly was not described before. Here, using three different approaches for exosomes isolation: commercial kit, nickel-based isolation, and ultracentrifugation methods and various mammalian cell lines, we elucidated the mechanisms responsible for APE1 secretion. We demonstrated that APE1 p37 and p33 forms are actively secreted through extracellular vesicles (EVs), including exosomes from different mammalian cell lines. We then observed that APE1 p33 form is generated by proteasomal-mediated degradation and is enzymatically active in EVs. Finally, we revealed that the p33 form of APE1 accumulates in EVs upon genotoxic treatment by cisplatin and doxorubicin, compounds commonly found in chemotherapy pharmacological treatments. Taken together, these findings provide for the first time evidence that a functional Base Excision Repair protein is delivered through exosomes in response to genotoxic stresses, shedding new light into the complex noncanonical biological functions of APE1 and opening new intriguing perspectives on its role in cancer biology.

The base excision repair (BER) pathway is responsible for repairing DNA lesions caused by alkylation damage or oxidative stress conditions due to endogenous or exogenous sources, including chemotherapy treatments (1, 2). The apurinic/apyrimidinic endodeoxyribonuclease 1 (APE1) is the main endonuclease that participates in the BER pathway and recognizes abasic sites on DNA and RNA in human cells (3, 4). In DNA, abasic sites can be generated by the action of glycosylases, such as OGG1, which are responsible for the recognition of modified bases and the hydrolysis of the N-glycosidic bond between the damaged base and the deoxyribose (5). Upon the action of APE1, a nick on the DNA backbone is introduced at the 5’ of the abasic site, generating an abasic dRP, which is subsequently processed by the downstream BER pathway enzymes, including Polβ, XRCC1, Polδ, and ligases (6). The generation of abasic sites in RNA is still a matter of debate, but its occurrence has been associated with profound biological consequences (7).

Besides its crucial role in the BER pathway, APE1 exerts several nonrepair functions, which have been characterized in the last 20 years (2, 8), such as: i) the redox regulation of transcription factors; ii) the control of redox homeostasis; and iii) the activity in metabolism and stability on coding and noncoding RNAs (ncRNAs) (9).

The multifunctional activity of APE1 depends on a modular structure characterized by three functional subregions: i) the N-terminal region, including the first 33–35 unstructured amino acidic residues, which is mainly involved in the stabilization of protein–protein or protein–RNA interaction (10); ii) the central region of the protein, comprising amino acid 35 to amino acid 127, which exerts the nonrepair activities based on redox mechanisms; and iii) the C-terminal domain, which entails the AP-endonuclease activity responsible for DNA repair (11). Interestingly, the 33–35 unstructured domain can be cleaved off by proteasomal-mediated mechanisms (12, 13), representing a mechanism to modulate APE1 different functions.

Biological evidence for the multifunctional activities of APE1 is supported by omics data, obtained in APE1 knock-down HeLa cells, showing significant variations in the expression of hundreds of genes, associated with: i) mitochondrial-mediated apoptosis; ii) dysregulation of the intracellular redox state, and iii) generation of membrane ruffling, as a consequence of a modification of the cytoskeleton (10).

APE1 can act as a coactivator or as a corepressor of gene transcription through redox-dependent (for this reason, APE1 is also called Ref-1, redox effector factor 1) and redox-independent mechanisms (14). In the redox-based mechanism, which involves cysteine residues susceptible to redox regulation (14, 15, 16), APE1 promotes the activation of transcription factors (such as NF-κB, AP1, HIF-1α, and STAT3) involved in chemoresistance responses, by modulating their redox state and promoting their activation upon different stimuli (10, 17).

More intriguingly, and largely unexplored yet, is the unsuspected role of APE1 in RNA metabolism, which plays a significant impact on microRNAs (miRNAs) expression (10, 18). We recently demonstrated that APE1 is involved in controlling miRNAs stability and regulation, during oxidative stress conditions, through the interaction with DROSHA (18). An alteration of miRNAs expression profile was observed in human osteosarcoma cells upon APE1 knock-down (19). Analysis of promoters regulating a list of miRNAs affected by APE1 downregulation and involved in cell growth, cell signaling, and cancer development clearly showed the presence of putative binding sites for transcription factors, such as NF-κB, p53, HIF-1α, AP1, and c-Myc, redox-regulated by APE1. These observations suggested that APE1 could indirectly regulate gene expression through miRNAs transcriptional control of target genes (19). It is also emerging that the different functions of APE1 are modulated by distinct interactomes, which redirect the protein activities to different substrates (20).

These findings provided the molecular basis for considering APE1 as a prognostic factor in several tumors such as: ovarian, hepatic, pulmonary, and neurologic cancers (8, 9, 21, 22), in which the protein is often overexpressed in both the nuclear and cytoplasmic compartments. For all these reasons, it can be hypothesized that a dysregulation of APE1 expression may contribute to tumor development and progression through mechanisms independent from the canonical DNA repair function (18). APE1 is also emerging as an important predictive factor being associated with chemoresistance phenomena due to its positive role in expression of cancer resistance genes, such as MDR1 and others (23, 24).

Interestingly, APE1’s presence in the extracellular milieu is a novel unsuspected biological aspect of the protein, yet poorly characterized (25, 26, 27). The link between APE1 amount in sera and tumor progression and chemoresistance was proved in a previous study performed on non-small-cell lung cancer (NSCLC) patients, in which the levels of serum APE1 (sAPE1) resulted significantly more elevated than in healthy controls and were associated with a worse progression-free survival (28). Therefore, sAPE1 is actually considered as a novel biomarker for the prognosis of NSCLC (28). Confirmatory results, along these lines, were recently obtained by our laboratory in a cohort of hepatocellular carcinoma (HCC) patients (29), in which we found that sAPE1 levels correlated with poor prognosis and were able to discriminate between cancer patients and cirrhotic or healthy donors (29). The presence of this protein in sera of patients is not solely restricted to cancer diseases but also in inflammatory models, such as coronary artery disease (30) and endotoxemia (31).

The biological function of secreted APE1 is still completely unknown. An intriguing hypothesis considers that it might act as a paracrine molecule in triggering cell-to-cell communication, important for the local tissue microenvironment inflammatory response (25, 26, 27, 29).

Also evidences on the mechanisms responsible for APE1 secretion are lacking, even though the importance of the acetylation, occurring on specific lysine residues sited in the first 33 N-terminal portion of the protein, has been highlighted in cells treated with the histone deacetylase inhibitor trichostatin A (TSA) (25). However, it seems reasonable that APE1 secretion might derive from extracellular vesicle formation *via* endosomal sorting complex (ESCRT), due to the protein lacking of a classic secretory signal peptide (28). This pathway is responsible for the biogenesis and maturation of multivesicular bodies (MVBs), composed of many intraluminal vesicles (ILVs), which are released in the extracellular milieu as exosomes. ILVs formation can occur through several mechanisms, and information about the regulation of these processes and the possible differences between the promoted cargo selection is still missing (32).

In this work, by using different biochemical techniques and several tumor and nontumor cell lines, it was demonstrated that secreted APE1 is exosome-associated. We also found that APE1 secretion is an active mechanism, exacerbated by genotoxic treatments, and that the secreted protein is enzymatically active. Altogether, these findings add new light in understanding the complex biological roles of APE1 in mammalian cells.

## Results

### APE1 protein is secreted through exosomes in EVs from HCC cancer cell line

Recent evidence obtained from human monocytes, upon inflammatory challenges, indicated the presence of APE1 in the extracellular milieu and possibly associated with EVs (26). In our previous work, we found that APE1 may be released in sera of HCC patients, with significantly higher levels in comparison to healthy donors (29). Thus, we evaluated whether APE1 could be secreted through small EVs, *e.g.*, exosomes, using the JHH-6 HCC cell line.

**Figure 1:**
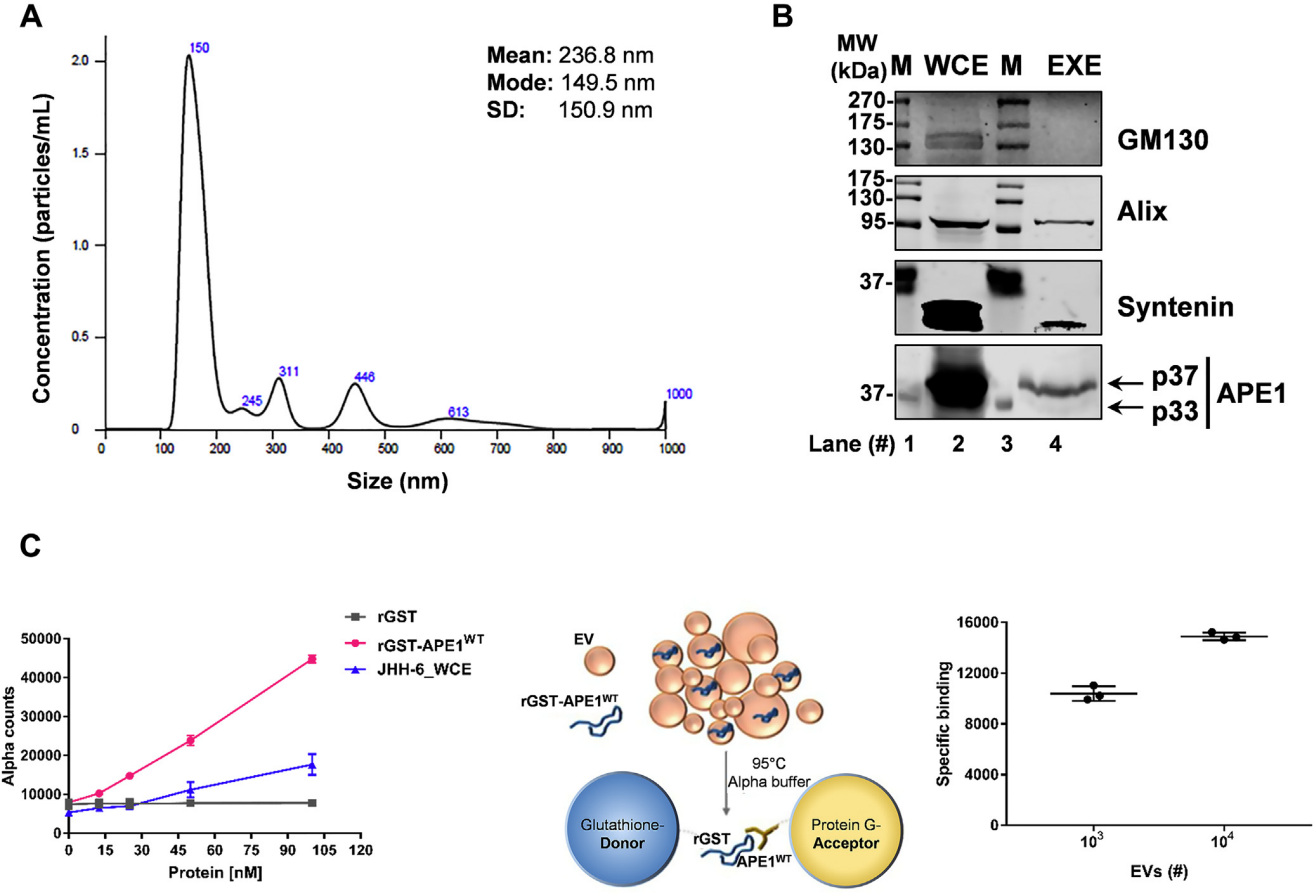
**EVs characterization and APE1 protein content analysis in JHH-6 cells:***A*, average size distribution curve determined by NTA of EVs derived from JHH-6 conditioned medium. The average concentration of vesicles was plotted against their size. The mean mode sizes and standard deviations are reported nearby the curve. *B*, western blotting analysis performed in JHH-6 WCE and EXE for APE1 detection (predicted molecular weight 37 kDa). APE1 p37 and p33 forms were observed in EXE. WCE were used as a positive APE1 control (*lower panel*). Golgi apparatus protein GM130 (apparent molecular weight 130 kDa) was assayed as exosome negative marker, Alix (apparent molecular weight 95 kDa) and Syntenin (apparent molecular weight 32 kDa) were assayed as exosome positive markers. *C*, AlphaScreen assay titrating the recombinant rGST-APE1 protein using Glutathione Donor beads and Protein A/G Acceptor beads in the presence of anti-APE1 antibody. The signal of the recombinant protein was detected in NBI-isolated vesicles upon a 95 °C heating step. The specific fluorescence counts, indicating a high signal to background ratio, were normalized to background after subtraction of the aspecific binding when considered at two EV concentrations in the scatter plot.

First, we isolated EVs from JHH-6 serum-free media using Exosome isolation kit (Invitrogen) and characterized the quality of the preparations obtained, as indicated in Material and Methods. JHH-6 cells were kept in culture for 24h under serum starvation conditions and then EVs were isolated. Purified particles were analyzed by Dynamic Light Scattering (DLS), using the NanoSight instrument, and nanoparticle tracking analysis (NTA). Figure 1*A* shows a representative distribution of vesicles with a mean concentration of 1.39e + 09 ± 0.00e + 00 particles/ml, with a mode of 149.5 nm and an average of 237 nm, ranging from ∼50 to ∼550 nm of the main populations. The morphology of the recovered EVs, which peaked at 150 nm in size, was assessed by Transmission Electron Microscopy, confirming the presence of round-shaped vesicles with a mean diameter around 80 nm (data not shown). Then, we performed a protein analysis of exosomal markers. Western blot analysis of EVs (EXE) confirmed the presence of Syntenin and Alix proteins, luminal markers of EVs from endosomal origin, in contrast to the Golgi apparatus protein GM130, used as negative control (33, 34, 35) (Fig. 1*B*). Therefore, this preliminary characterization confirmed that EVs included exosomes.

Furthermore, we analyzed the presence of APE1 in EVs. As shown in Figure 1*B*, EXE from JHH-6 cells confirmed the presence of a specific band of 37 kDa (APE1 p37) corresponding to the full-length APE1 protein, as well as a faint band corresponding to the truncated form of APE1 at 33 kDa, indicated as APE1 p33. Notably, by using an antibody specific for the N-terminal region of APE1, we demonstrated that this truncated form corresponded to the NΔ33 one (Fig. S1 and data not shown), which loses the first 33 amino acid residues as a consequence of proteolysis, as already described in previous papers (29, 36, 37).

To quantify APE1 protein levels in EVs, we used an EV isolation procedure recently published (38), *i.e.*, nickel-based isolation (NBI), allowing to minimize particle aggregation in solution and suitable for combination with homogeneous assays (39). We applied NBI to the media of JHH-6 cells to isolate EVs and then combined an amplified luminescence proximity homogeneous assay (alpha). We designed the alpha assay using purified and quantified recombinant Glutathione S-transferase-APE1 (rGST-APE1^WT^) (Fig. S2). The alpha assay was performed using Glutathione-Donor beads and Protein G-Acceptor beads recognizing the anti-APE1 antibody. We tested the detection of the recombinant protein by titrating the purified rGST-APE1^WT^ protein, protein lysates from JHH-6 cells, and purified GST alone as further control (Fig. 1*C*). We then exploited the recombinant protein as a reference substrate in the alpha assay. In this setting, we sensitively and specifically detected the rGST-APE1^WT^ in the low nanomolar range compared with GST alone (Fig. 1*C*). In Fig. 1*C*, 100 nM of rGST-APE1^WT^ represents the hook point (Fig. S3). Subsequently, we isolated vesicles from JHH-6 cells by NBI (38) and applied the alpha assay using a range of 10^2^–10^5^ vesicles, as determined by the Tunable Resistive Pulse Sensing (TRPS) technology and qNANO instrument with an NP250 nanopore (the applied stretch gave a size window of approximately 100–800 nm for the majority of vesicle subpopulations recovered). We only detected specific signals using EVs exposed to 90 °C denaturation for 5 min before titrating them (Fig. 1*C*), indicating that recombinant rGST-APE1 is enclosed within vesicles. According to the standard curve, obtained using the purified rGST-APE1, we calculated the remarkable amount of 1.27 ng of protein every 10^3^ EVs having a size range between 100 and 700 nm, shown in Fig. S4, and a recovery in the range of 10^9^ vesicles/ml.

**Figure 2:**
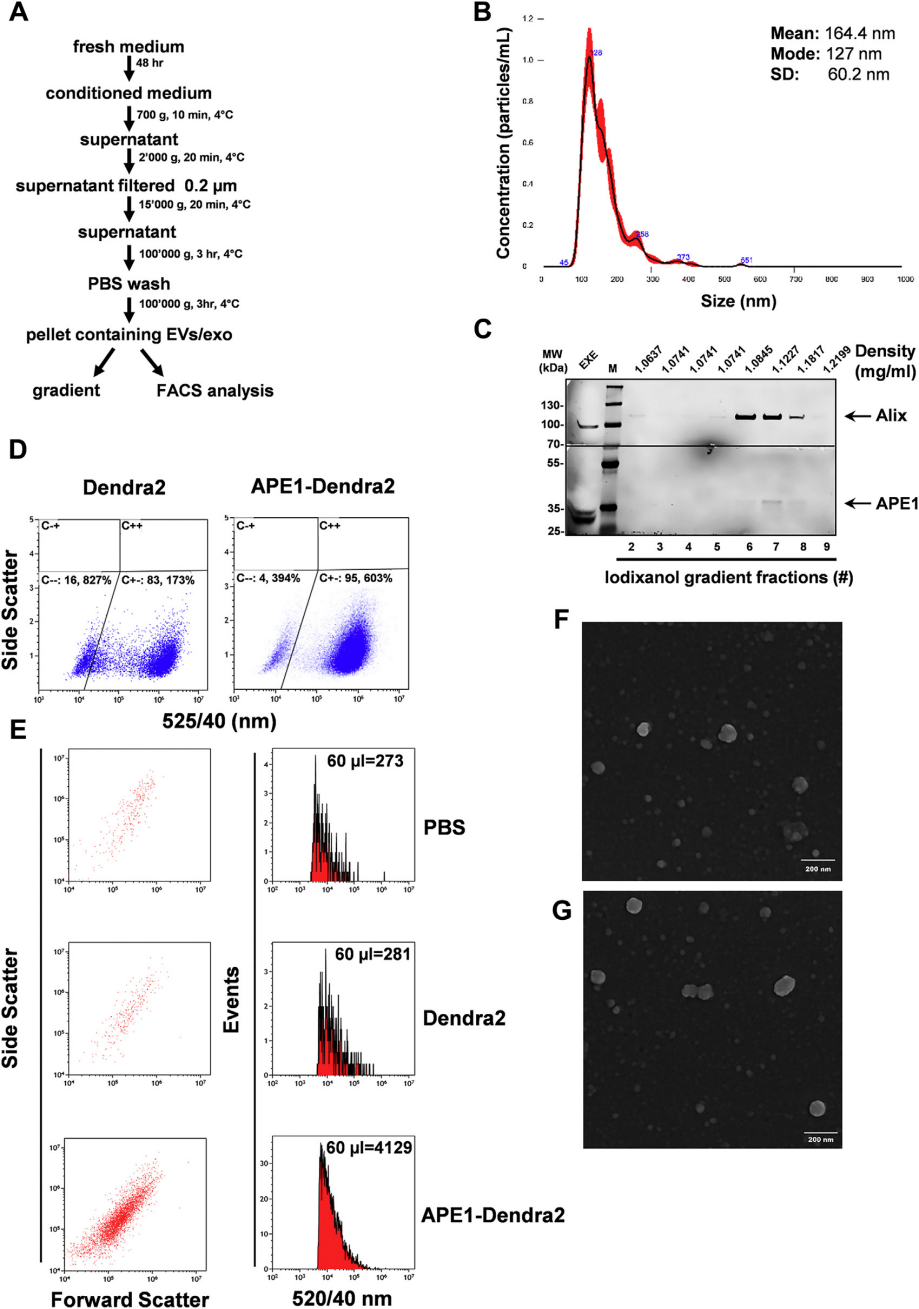
**APE1 is associated to exosomes isolated by ultracentrifugation:***A*, workflow of the processing of cell-conditioned medium by ultracentrifugation-based technique. *B*, size distribution of sEV/exo crude pellet was performed by nanoparticle tracking analysis (NTA). Numbers on the graph represent the mode (most often occurring dimension). Note that the main peak corresponds to a mode of 126 nm. *C*, Western blotting analysis of APE1 in iodixanol gradient fractions; exosomes (60 μg) obtained by ultracentrifugation of CM (48 h) from JHH-6 cells were loaded at the bottom of iodixanol gradient and subjected to ultracentrifugation for 19 h. An equal volume of each fraction was analyzed for APE1 and Alix expression. The molecular mass (MW), expressed in kDa, is shown on the *left side* of the image. *Horizontal black line* in correspondence to 70 kDa indicates the cutting site carried out for blotting the membrane respectively with Alix (high) and APE1 antibodies. Fraction densities were determined by refractometry. *D*, flow cytometry analysis of Hela- Dendra2 (*left dot plot*) and Hela-APE1-Dendra2 (*right dot plot*) transfected cells. The Dendra2 positive cells were identified by plotting fluorescence at 525/540 nm versus side scatter channel. The Dendra2 positive cells were determined comparing the mock-transfected HeLa cells negative for Dendra2 expression (data not shown). *E*, flow cytometry analysis of APE1-Dendra2 in exosomes. Exosomes isolated by ultracentrifugation from CM of cells described in (*D*) were resuspended with same volume of PBS. The number of events registered in 60 μl is shown in the histogram column (*left*). PBS (60 μl) was analyzed to establish the amount of background noise. The *dot plot* columns on the left show forward and side scatter of the acquired exosomes. *F*, electron micrographs of exosomes obtained from HeLa-Dendra2 cells (*F*) and HeLa APE1-Dendra2 cells (*G*). Bars, 200 nm.

To definitively assess whether the association of APE1 to EVs was ascribable to exosomes, we combined biochemical and cytofluorometric approaches on EVs isolated using an ultracentrifugation-based protocol (40) (Fig. 2*A*), which represents a gold standard procedure to isolate small vesicles including exosomes. The crude pellet was first characterized for size distribution by using the Nanosight technology. The results showed the presence of a main population with a mode of 127 nm and a mean value of 164, 4 nm in accordance with the expected exosome size (Fig. 2*B*). In the biochemical approach, we evaluated the co-occurrence of density and distribution of APE1 and the exosome specific marker, *i.e.*, Alix, on an iodixanol gradient. Thus, cell-conditioned medium was subjected to ultracentrifugation and the resulting pellet was loaded onto 10–40% iodixanol gradient and centrifuged. The obtained fractions were then analyzed for APE1 and Alix content by western blotting. Results indicated the effective codistribution of APE1 and Alix in fractions with a density comprised between 1.08 and 1.18 g/ml, corresponding to the one specific for exosomes (39) (Fig. 2*C*). The mobility of APE1 and Alix proteins in SDS PAGE was altered by iodixanol’s presence.

In the final cytofluorometric approach, we took advantage from an engineered cell line expressing the fluorescent APE1-Dendra2 construct available in the Lab (41). Cells were first analyzed for APE1-Dendra2 and Dendra2 expression, which showed similar fluorescence (Fig. 2*D*). Then, we evaluated, by a direct cytofluorometric analysis, the presence of APE1-Dendra2 in isolated exosomes. Results showed the presence of a single fluorescent population (Fig. 2*E* *lower panel*), with a minimal background noise on 520/40 channel, as observed under PBS condition (Fig. 2*E* *upper panel*). Interestingly, in the control exosomes secreted from cells expressing Dendra2, similarly to PBS condition, only few events could be registered (Fig. 2*E* *middle panel*), thus indicating the specific association of APE1 with exosomes. A final ultrastructural analysis by scanning electron microscopy confirmed that in both exosome samples analyzed by FACS, intact round-shaped vesicles of 50–100 nm size were present (Fig. 2, *F* and *G*).

Overall, these data indicate that APE1 is present in EVs in association with exosomes.

### p37 and p33 APE1 forms are secreted in exosomes of different mammalian cell lines

**Figure 3:**
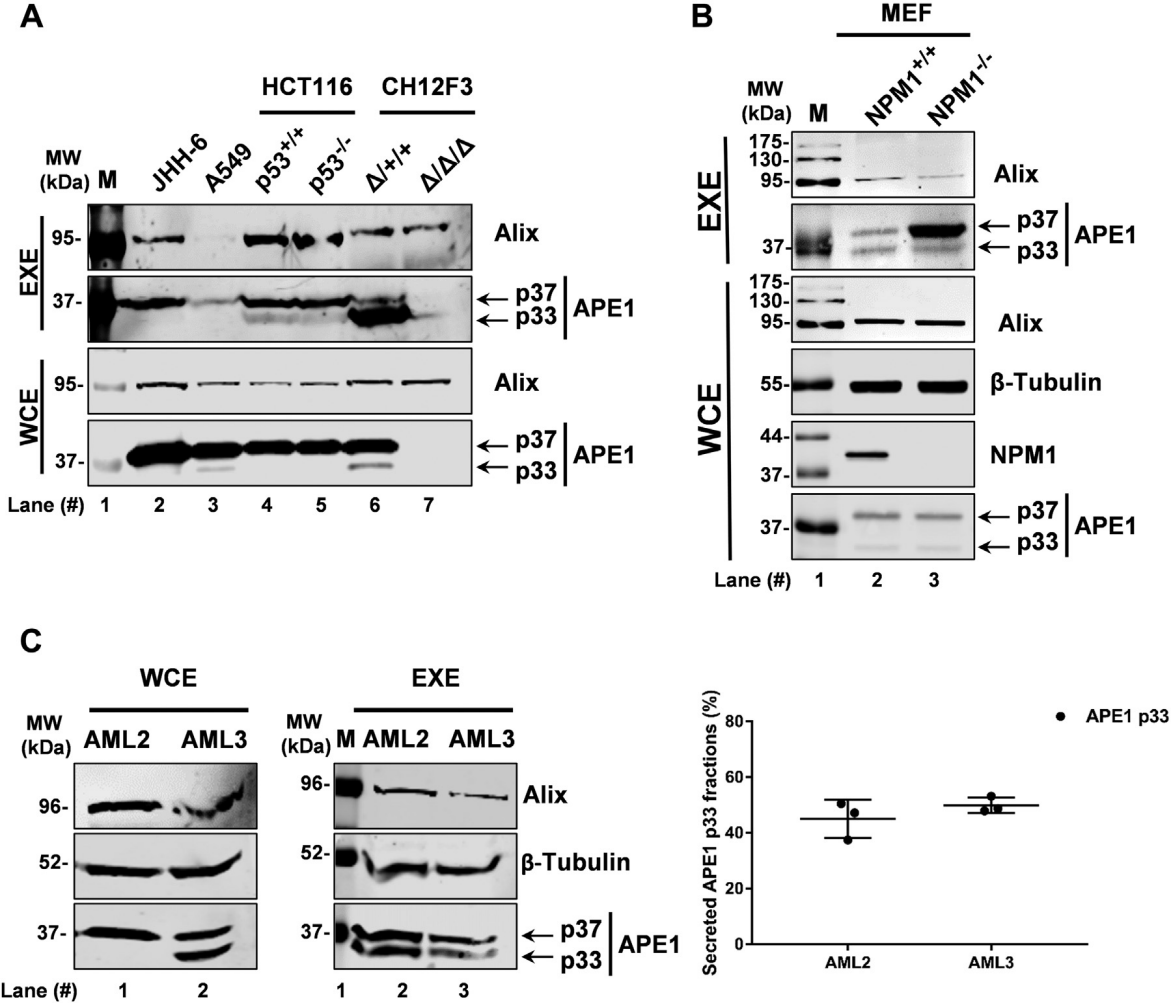
**APE1 EVs secretion in a panel of mammalian cell lines:***A*, western blotting analyses for APE1 detection carried out in EXE of JHH-6, A549, HCT116^P53+/+^, HCT116^p53-/-^, CH13F3^Δ/+/+^, and CH12F3^Δ/Δ/Δ^ cells. Both APE1 p37 and p33 forms have been detected. Alix was used as a loading control. WCE were analyzed as a positive control of APE1 expression. The molecular mass (MW) expressed in kDa is shown on the *left side* of the image. *B*, western blotting analyses for APE1 detection carried out in EXE of MEF^NPM1+/+^ and MEF^NPM1-/-^ cells. Both APE1 p37 and p33 forms have been detected. Alix was used as a loading control. WCE were analyzed as a positive control of APE1 expression and β-Tubulin (apparent molecular weight 52 kDa) was detected as loading control. Western blotting analysis for NPM1 detection was carried out as a control for cells genotype. The molecular mass (MW) expressed in kDa is shown on the *left side* of the image. *C*, western blotting analyses for APE1 detection in WCE and EXE derived from OCI-AML2 and OCI-AML3 cell lines. β-Tubulin (apparent molecular weight 52 kDa) and Alix (apparent molecular weight 95 kDa) detection was carried out as loading controls. APE1 p37 and p33 are indicated by *arrows*. The molecular mass (MW) expressed in kDa is shown on the *left side* of the image. The percentages of exosomal APE1 p37 and p33 forms in OCI-AML2 and OCI-AML3 cell lines are indicated in the scatter plot on the *right panel* and in Fig. S5. Data are expressed as mean ± SD of three independent replicas.

In order to test whether the secretion of APE1 through exosomes is a common feature of different mammalian cell lines, we performed EXE analysis in lymphocyte mouse isogenic cell lines, *i.e.*, the non-tumor CH12F3^Δ/+/+^ and CH12F3^Δ/Δ/Δ^, expressing or not APE1 through genetic deletion, respectively (42). The use of these isogenic cell lines was also instrumental to further demonstrate the identity of the APE1 band recognized by the antibodies used in our study. Using these two cell lines, we found that APE1 was indeed exclusively present in the exosomes obtained from the CH12F3^Δ/+/+^ cells expressing APE1, with a significant presence of the p33 form (Fig. 3*A*). Moreover, these data suggested that the release of APE1 in exosomes was not exclusively limited to JHH-6 cells. Indeed, testing exosomes obtained from: A549 lung tumor cell line, HCT116^p53+/+^ and HCT116^p53-/-^ colon carcinoma isogenic cell lines confirmed the general nature of this phenomenon (Fig. 3*A*). Moreover, in order to confirm that the secretion of APE1 is common in vesicles from non-cancer cell lines, we used two mouse embryonic fibroblast (MEF) cell lines expressing or not Nucleophosmin 1 (NPM1) (43), a known APE1–protein interacting partner (44). Data obtained (Fig. 3*B*) enforced the presence of APE1 in exosomes as a general occurrence in all mammalian cell lines tested. Interestingly, we observed that the amount of the p37 and p33 protein forms was different between cell lines tested. These data clearly demonstrated that both the p37 and p33 forms of the APE1 protein may be released, to different extents and ratios, in exosomes of mammalian cell lines of different origin.

Based on our previous characterization of the p33 form in cancer cells from hematopoietic origin, *i.e.*, acute myeloid leukemia (36), we tested the distribution of APE1 in EXE of OCI-AML3 (expressing the mutated NPM1c+ protein) and OCI-AML2 control cells (45). These two cell lines represent a suitable model for this analysis, as OCI-AML2 cells show normal subcellular expression of APE1 protein with a prominent nuclear distribution and no significant trace of theAPE1 p33 form (36). Interestingly, the OCI-AML3 cell line harbors a driver mutation of the NPM1 gene, called NPM1c+, which causes APE1 cytoplasmic localization and consequent BER impairment (36). In OCI-AML3 cells, the APE1 protein is relocalized within the cytoplasm, where it undergoes proteolytic degradation of the first 33 amino acids at the N-terminal sequence (12) (Fig. 3*C*-*left panel*). When the APE1 p37 and p33 protein content in EXE, obtained from OCI-AML2 and OCI-AML3 cells, was examined, we observed that either of forms is released by both cell lines, as shown in Figure 3*C* (*middle panel*). In particular, in the OCI-AML2 cells, the amount of total secreted APE1 (comprising both the p37 and p33 APE1 forms) was higher than that secreted by the OCI-AML3 cells. Interestingly, the ratio between the p37 and p33 APE1 forms was comparable between EXE of both cell lines (Fig. 3*C* *right panel* and Fig. S5). These experiments indicate that while the OCI-AML2 cells, in contrast to the OCI-AML3 cells, do not show any p33 protein form in WCE, as previously observed (36), the p33 form was present in the vesicle extracts of both cell lines at comparable levels. These data could suggest that the secretion efficiency of the APE1 p33 form by OCI-AML2 is higher than that of the OCI-AML3. Alternatively, this data could indicate that a common proteolytic event, which is absent in the cytoplasm of the OCI-AML2 cells, may also occur in exosomes.

### APE1 p33 form in exosomes is generated through proteasomal-mediated degradation

**Figure 4:**
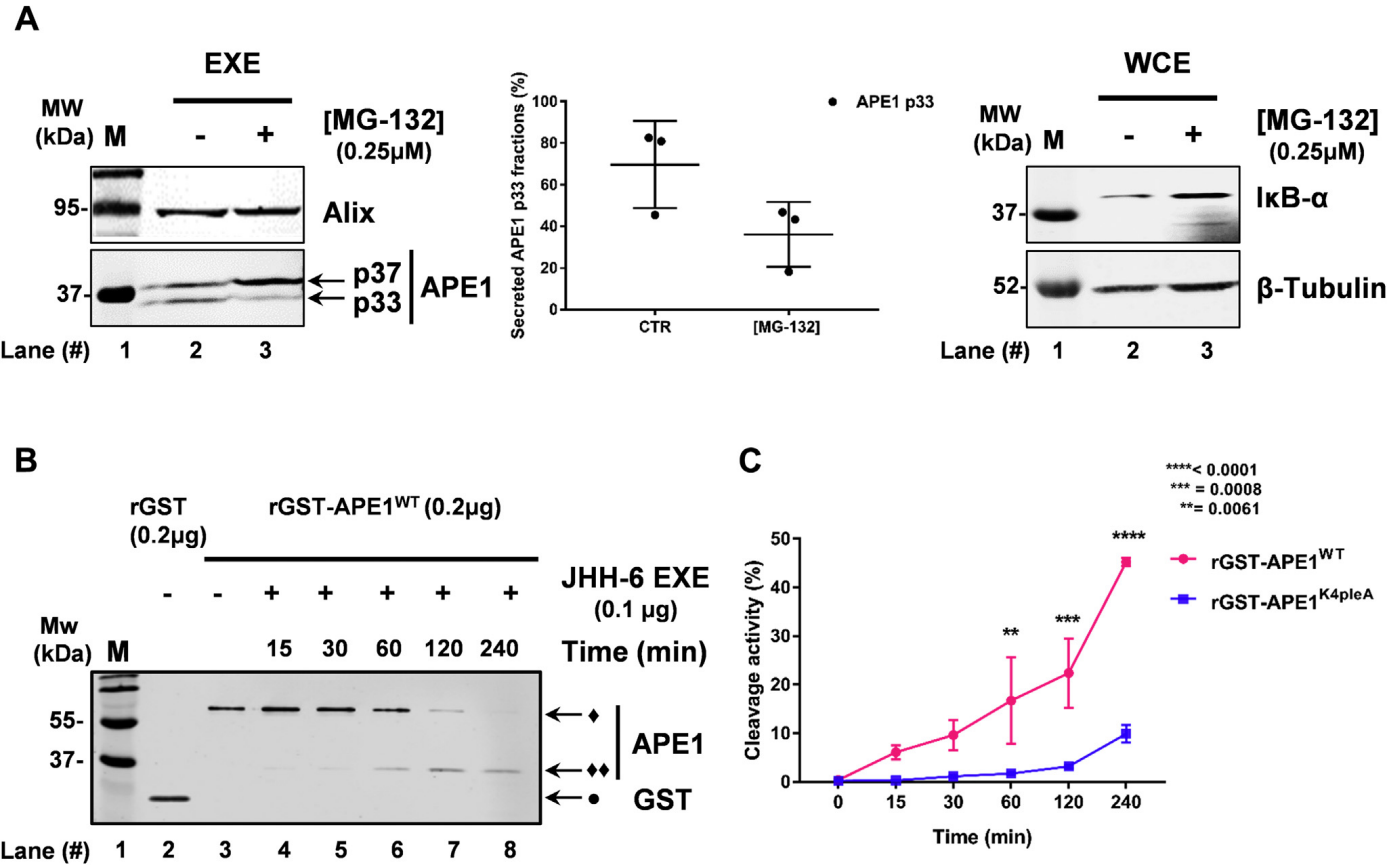
**APE1 proteolytic activity characterization in exosomes:***A*, western blotting analyses for APE1 p37 and p33 detection in EVs derived from JHH-6 MG-132 treated cells and the respective untreated CTR cells (*left panel*). Alix (apparent molecular weight 95 kDa) was used as loading control. The percentage of exosomal APE1 p37 and p33 forms in both CTR and MG-132 JHH-6 cells has been obtained by densitometry analysis, and it is indicated in the scatter plot (*middle panel*) and in Fig. S6. Western blotting analysis for IκB-α (apparent molecular weight 38 kDa) detection was performed in CTR and MG-132 treated JHH-6 WCE as a confirmation of proteases inhibition. The molecular mass (MW), expressed in kDa, is shown on the *left side* of the image. Data are expressed as mean ± SD of three independent replicas. *B*, EVs possess proteases able to cleave rGST-APE1 protein. *In vitro* proteolytic activity exerted by EXE (0.1 μg) upon rGST-APE1^WT^ (0.2 μg) (apparent molecular weight 65 kDa). The reactions were performed at 37 °C for the indicated times and the substrates (♦) and the products (♦♦) of reactions have been detected by Western blotting using APE1 antibody (*left panel*). rGST (0.2 μg) (apparent molecular weight 29 kDa) indicated as (•) has been detected by western blotting using GST antibody. *C*, graph illustrating the comparison of EXE cleavage activity upon rGST-APE1^WT^ and rGST-APE1^K4pleA^. Data are expressed as mean ± SD of three independent technical replicas. ∗*p* < 0.05, ∗∗*p* < 0.01, ∗∗∗*p* < 0.001, ∗∗∗∗*p* < 0.0001.

Recent published data from our lab clearly demonstrated that the cleavage of APE1 N-terminal region, giving rise to the p33 form of the protein, is, at least in part, caused by cytoplasmic proteasomal complex activity (12). In order to evaluate whether the proteasomal pathway can be also involved in the accumulation of the p33 form in exosomes, we blocked the proteasome-mediated degradation with MG-132 inhibitor (46) in JHH-6 cells. Figure 4*A* and Fig. S6 show a significant reduction of the p33 form only upon MG-132 treatment, demonstrating that the APE1 truncated form, accumulated in the exosomes, is substantially contributed by an intracellular proteasomal activity. The efficacy of MG-132 treatment was proved by evaluating the accumulation of IκB-α (47) in WCE, as shown in Figure 4*A*, *right panel*.

Proteasomal components can be actively secreted in exosomes (48, 49, 50). We therefore tested whether exosomes could contribute to APE1 N-terminal cleavage. For this purpose, JHH-6 EXE was tested for its proteolytic activity using rGST-APE1^WT^ protein, as a substrate, in kinetics experiments. The reactions were carried out for different times at 37 °C. rGST was used as an additional control of gel migration. Upon reaction, the samples were denatured and separated on SDS-PAGE. As shown in Figure 4*B*, JHH-6 EXE showed a considerable time-dependent cleavage activity on rGST-APE1^WT^ protein, as demonstrated by western blot analysis with an antibody specific for APE1 protein (Fig. 4*B*). Moreover, analysis of the blot with an antibody specific for the GST protein (Fig. S7) showed that exosomes’ proteolytic activity was able to remove the GST tag from the rGST-APE1^WT^ fusion protein used as a substrate in these assays, considering that the obtained products showed the expected molecular weight of the p37 form. In order to demonstrate the occurrence of cleavage at the N-terminal region of APE1, we used a purified recombinant APE1 mutant (Fig. S2) bearing four Lys (K27, 31,32, 35) substituted with Ala to abolish the sites essential for APE1 proteasomal cleavage, as previously demonstrated (12). Figure 4*C*, data not shown, and Fig. S7 clearly show that Lys to Ala substitution significantly hampered the proteolytic activity by exosomes, demonstrating that the observed activity occurs at the level of the region 31–35 of APE1, as previously demonstrated within cells. These data indicate that APE1 cleavage involves the 33N-terminal region of the protein and implies a role of the proteasome in the cleavage step.

### APE1 protein from exosomes extracts is enzymatically active

**Figure 5:**
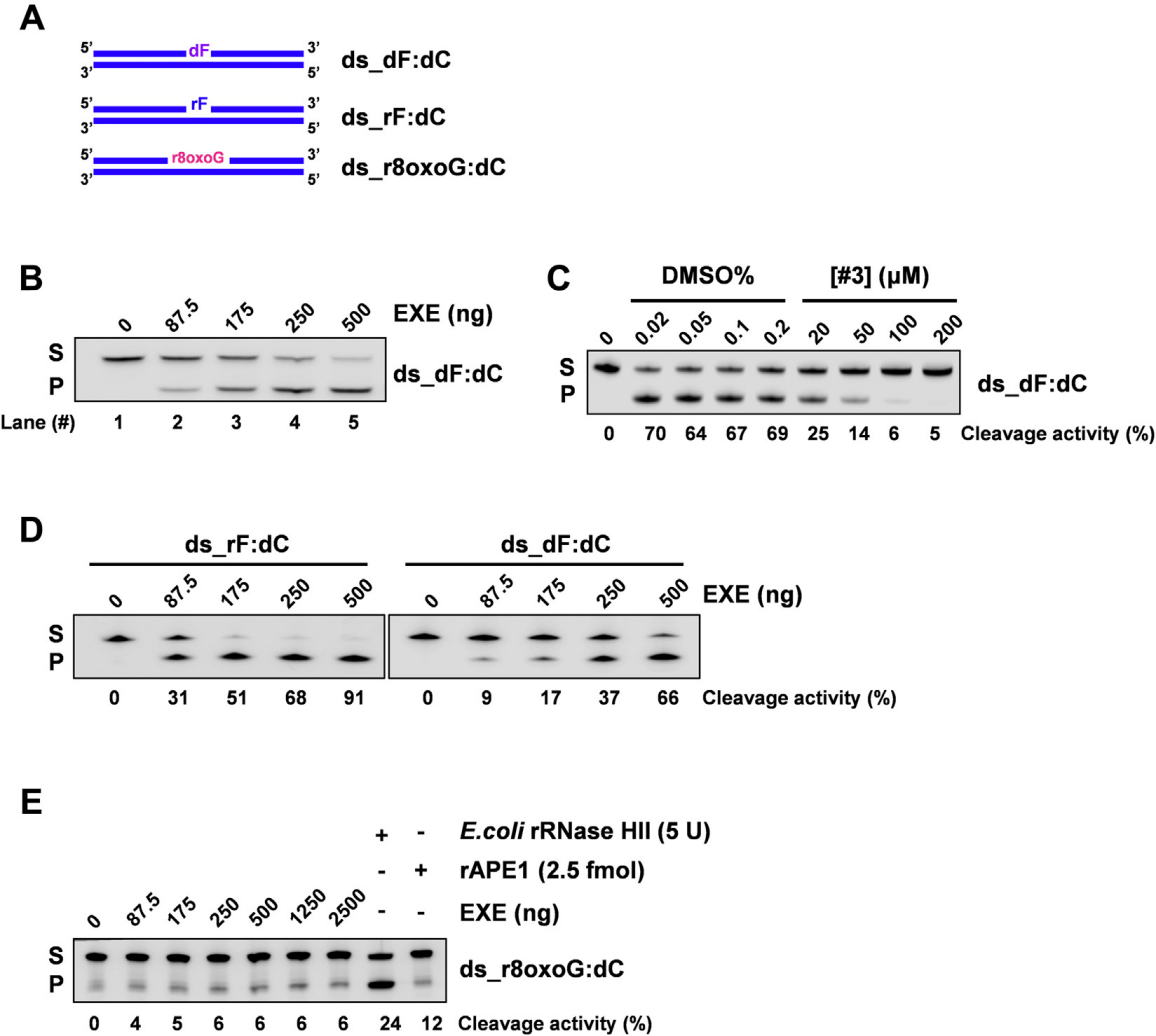
**APE1 in exosomes is enzymatically active:***A*, schematic representation of dsDNA 25-mer substrates generated by annealing of ss_dC complementary oligonucleotide (5’-GTTCAGGCCTAACACTACCGGATCC-3’) with IRDye fluorophore labelled ssDNA oligonucleotide (5’-GGATCCGGTAGTGTTAGGCCTGAAC-3’), where the G in the 13th position is modified in deoxy-tetrahydrofuran (dF) (*light purple*) or ribo-tetrahydrofuran (rF) (*light blue*) or oxidized ribo-guanosine (r8oxoG) (*pink*). The 5′ and 3′ ends of each DNA strand are indicated. *B*, JHH-6 EXE-containing APE1 possesses endonucleolytic activity on the canonical abasic dsDNA substrate. Representative denaturing polyacrylamide gel of EXE incision on ds_dF:dC oligonucleotide. The quantity of EXE used, expressed in ng, is shown on the top of the figure. The reaction was performed for 15 min. Data are the mean of three independent technical replicas. *C*, representative denaturing polyacrylamide gel of EXE (250 ng) incision on ds_dF:dC oligonucleotide for 15 min following pretreatment of EXE at different concentrations (μM) of APE1 inhibitor #3 for 15 min. Different concentrations of DMSO (%) were used as control. The mean cleavage activity expressed in percentage is indicated below the *panel*. Data represent the mean of three independent technical replicas. *D*, JHH-6 EXE-containing APE1 possesses endonucleolytic activity on abasic ribonucleotides embedded in dsDNA. Representative denaturing polyacrylamide gel of EXE incision on ds_rF:dC oligonucleotide. The quantity of EXE used, expressed in ng, is shown on the top of the figure. The reaction was performed for 15 min. ds_dF:dC oligonucleotide was used as positive control. In parallel, representative denaturing polyacrylamide gel of EXE incision on ds_dF:dC oligonucleotide is shown in the *right panel.* Data represent the mean of three independent technical replicas and the mean cleavage activity expressed in percentage is indicated below *panels*. *E*, JHH-6 EXE-containing APE1 possesses a weak endo- and exo-nucleolytic activity on oxidized ribo- G embedded in dsDNA. Representative denaturing polyacrylamide gel of EXE incision on ds_r8oxoG:dC oligonucleotide. The amount of EXE used, expressed in ng, is shown on the top of the figure. The EXE-containing APE1 activity was compared with the recombinant APE1 (rAPE1) activity on the same substrate at the indicated dose. The reaction was performed for 30 min. The mean cleavage activity expressed in percentage is indicated below the panel. Data represent the mean of three independent technical replicas.

We then tested whether exosomal-APE1 was enzymatically active on different kind of DNA substrates (51) bearing an abasic deoxyribonucleotide in dsDNA mimicked by a tetrahydrofuran residue (ds_dF:dC), an abasic ribonucleotide embedded in DNA (ds_rF:dC), and an r8oxoG embedded in DNA (ds_r80x0G:dC), as depicted in Figure 5*A*. For this purpose, we used increasing amount of EXE to test the APE1 endonuclease activity. First of all, we checked exosomal APE1 activity on canonical ds_dF:dC (Fig. 5*B*) by using classical endonuclease assay (51). Results demonstrated that APE1 from EXE was competent to process the canonical substrate ds_dF:dC in a dose-dependent manner (Fig. 5*B*). Then, in order to demonstrate whether the enzymatic activity of EXE was exclusively ascribed to APE1, an endonuclease assay was performed after treating EXE with different doses of compound #3, a specific inhibitor of APE1 endonuclease activity (44, 52) (Fig. 5*C*). This experiment confirmed that APE1 activity in the EXE is the only endonuclease activity on abasic dsDNA present within exosomes. Next, we tested whether APE1 was able to process an abasic ribonucleotide embedded in dsDNA (ds_rF:dC), similarly to what we recently found for nuclear APE1 (51). Enzymatic assays clearly showed that exosomal APE1 was able to efficiently process this substrate, showing an activity even higher than that of the one observed for the ds_dF:dC (Fig. 5*D*). Then, we tested the EXE activity on a noncanonical substrate through an NIR activity, resembled by a dsDNA of the same sequence as above, but containing an oxidized ribonucleotide embedded in dsDNA in the middle (ds_r8oxoG:dC), as we recently showed (51). As positive controls of the incision on this substrate, we used recombinant APE1 (rAPE1), to have a simultaneous evaluation of the expected products, and also *Escherichia coli* rRNase HII, which possesses a stronger 5’ endonuclease activity on this substrate (53). Data obtained (Fig. 5*E*) showed that APE1 from EXE displays a weak NIR activity, similarly to recombinant purified protein, as we previously demonstrated (51). Overall, these experiments proved that exosomal APE1 is able to fully exert its enzymatic activities on different DNA substrates containing both canonical abasic sites and noncanonical (r8oxoG) substrates.

### Secretion of APE1 p33 form is stimulated by doxorubicin treatment in the JHH-6 cell line and by cisplatin treatment in A549 cells

**Figure 6:**
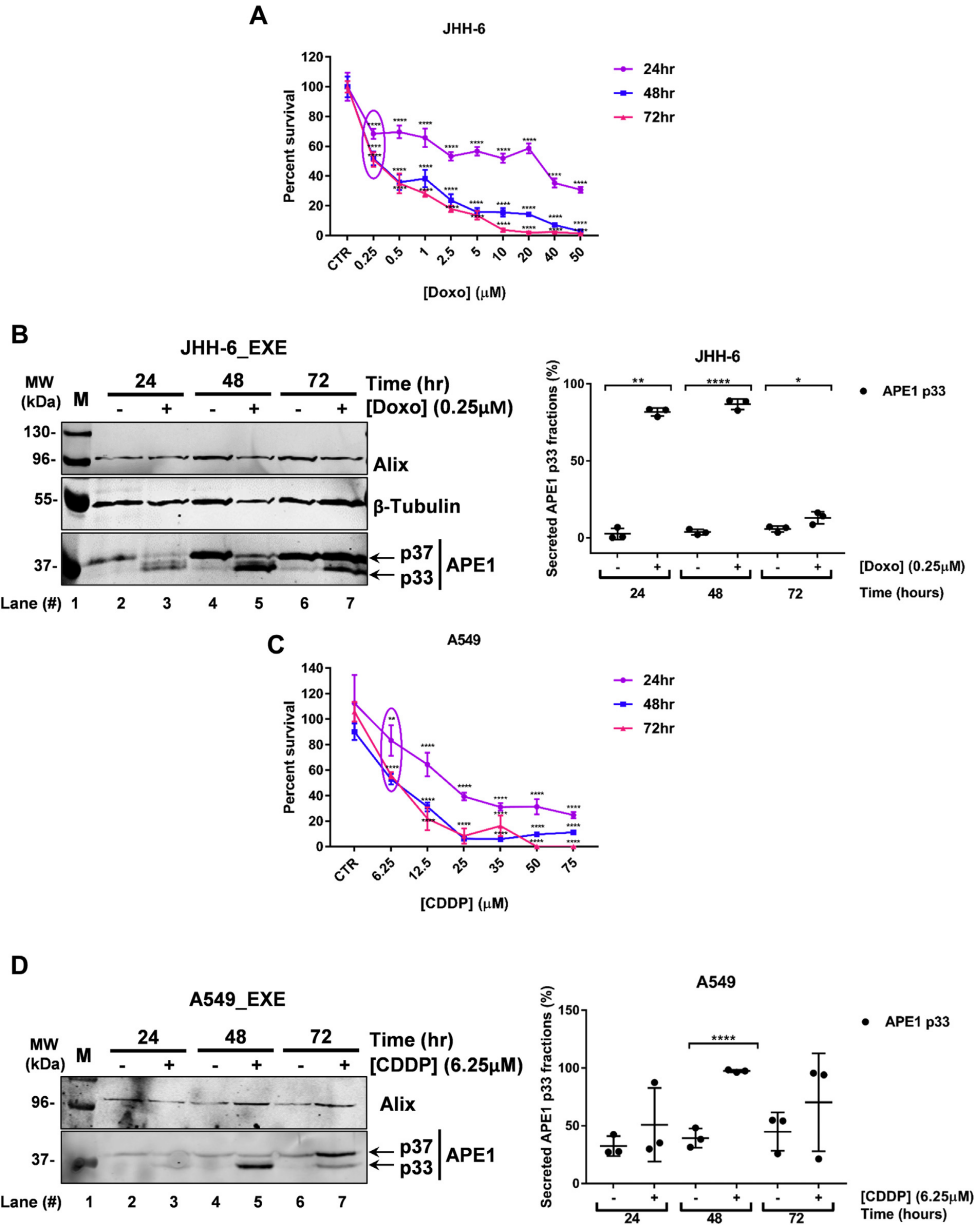
**Genotoxic stresses cause the modulation of APE1 p37 and p33 forms in EVs:***A*, cell viability analyses carried out in JHH-6 cell line treated for 24, 48, 72 h with the indicated doses of Doxorubicin, expressed in μM and the respective untreated cells indicated as CTR. Data are expressed as mean ± S.D of two independent replicas. *B*, analysis of APE1 accumulation and study of p37 and p33 distributions in JHH-6 EXE after treatments with sublethal concentration of Doxo at the indicated time points. JHH-6 cells were treated with 0.25 μM of Doxo for 24, 48, and 72 h. Media were collected and processed for obtaining EXE. EXE derived from JHH-6 untreated cells was analyzed as control (CTR). APE1 p37 and APE1 p33 patterns were analyzed by western blotting. Alix and β-Tubulin were detected as loading controls. Densitometry analysis was performed and the percentage of secreted APE1 p33 fraction was plotted in the scatter plot *on the right.* Row data of the percentage of APE1 p37 and p33 fractions are shown in Fig. S9. Data are expressed as mean ± SD of three independent replicas. *C*, cell viability analyses carried out in A549 cell line treated for 24, 48, 72 h with the indicated doses of CDDP and the respective untreated cells indicated as CTR. Data are expressed as mean ± S.D of two independent replicas. *D*, analysis of APE1 accumulation and study of p37 and p33 distributions in A549 EXE after treatments with sublethal concentration of CDDP at the indicated time points. A549 cells were treated with 6.25 μM of CDDP for 24, 48, and 72 h. Media were collected and processed for obtaining EXE. EXE derived from A549 untreated cells was analyzed as control (CTR). APE1 p37 and p33 patterns were analyzed by western blotting. Alix was detected as loading control. Densitometry analysis was performed and the percentage of secreted APE1 p33 fraction was plotted in the scatter plot *on the right.* Row data of the percentage of APE1 p37 and p33 fractions are shown in Fig. S11. Data are expressed as mean ± SD of three independent replicas. ∗*p* < 0.05, ∗∗*p* <0.01, ∗∗∗*p* < 0.001, ∗∗∗∗*p* < 0.0001.

We then wanted to test whether genotoxic stress may affect APE1 release through exosomes. Doxorubicin (Doxo) is an intercalating agent used in the treatment of HCC (54, 55). To evaluate whether APE1 release could be affected by genotoxic damage, we analysed APE1 secretion upon Doxo treatment. First, we determined the proper treatment conditions, represented by sublethal doses of drug, in order to correlate the effect of the treatment on EVs APE1 content with the viability of JHH-6 cells. As shown in Figure 6*A*, the treatment with Doxo, at the dose of 0.25 μM, was considered ideal because of exerting low toxicity upon 24 h of treatment and a toxicity of 50% after 48 and 72 h of treatment. Then, JHH-6 cells were treated for 24, 48, 72 h with Doxo at the concentration of 0.25 μM and the exosomes were isolated, as indicated before. In parallel, the analysis of WCE (Fig. S8) clearly demonstrated that Doxo treatment affected neither the expression of APE1 nor that of the Alix proteins. Interestingly, when we compared the expression of APE1 in exosomes, upon Doxo treatment, we noticed that the accumulation of the p33 form was increased with respect to control cells, as quantified in the scatter plot, in particular after 24 and 48 h of treatment (Fig. 6*B* *right panel* and Fig. S9).

In order to check if this trend was only related to a specific effect of Doxo treatment on exosomes release of APE1 or whether it was common to other genotoxic treatments, JHH-6 cells were treated with Cisplatin (CDDP), a DNA-cross-linking agent commonly used in the treatment of NSCLC (56, 57). Similar results were obtained using CDDP (data not shown), confirming that different genotoxic agents may promote the release of higher amounts of the APE1 p33 form compared with the p37 one in JHH-6 cells.

In order to have a more comprehensive view and extend the observation to other cancer cell lines, we repeated the experiment of CDDP treatment in the A549 lung cancer cell line. We chose the concentration of 6.25 μM, as shown in Figure 6*C*, because CDDP treatment at this concentration caused low toxicity at 24 h and a toxicity of about 50% upon 48 and 72 h of treatment, similarly to what we observed for JHH-6 cells. Then, we performed exosomes isolation from A549-conditioned media after 24, 48, 72 h of CDDP treatment, and we analyzed the amount of the APE1 p33 and p37 forms in WCE and EXE. Similarly to JHH-6 cells, no differences of the two APE1 protein forms were observed in WCE from A549, treated with CDDP, compared with untreated control cells (Fig. S10). Interestingly, we observed an accumulation of APE1 p33 form in EXE, compared with the p37 form, upon CDDP treatment of A549 cells (Figs. 6*D* and S11). Coomassie blue staining was carried out for A549 EXE loading control (Fig. S12).

Altogether, these findings corroborate the hypothesis that chemotherapeutic drugs may induce an exosomal accumulation of the APE1 p33 form compared with the p37 protein in different cancer cell lines, possibly suggesting the existence of different functions absolved by the p33 and p37 forms in EVs.

## Discussion

APE1 is a multifunctional protein that participates in DNA repair and in the regulation of gene expression. We have recently demonstrated its role in RNA processing, as a major AP endonuclease on abasic RNA (18, 24, 51) and on abasic ribonucleotides embedded in DNA (24, 51). Noteworthy, this unexpected activity on RNA possibly plays an important role in miRNAs processing, modulating gene expression under genotoxic stress conditions (18). Interestingly, elevated levels of APE1 protein in sera of different tumors, such as HCC and NSCLC, have been described (28, 29) and extracellular APE1 contribution in promoting chemoresistance, by affecting cell sensitivity to chemotherapy, was suggested (28, 58), allowing to hypothesize that sAPE1 could be considered as a prognostic and predictive biomarker in cancer. Whether sAPE1 could exert its biological function in association with extracellular vesicles was not fully addressed, yet. Indeed, APE1 was found associated, under basal conditions to EVs in human monocytes (26), but studies focusing on the constitutive vesicles association of APE1 in tumor cells, as well as a biochemical characterization of a secreted form, were completely lacking.

Our study was aimed at addressing this issue, and we demonstrated that, using several mammalian cell lines and different orthogonal assays, APE1 is secreted through EVs, particularly exosomes, and is enzymatically active. EVs represent a heterogeneous population comprising vesicles of different biogenesis, such as exosomes from the endosomes and microvesicles from plasma membrane budding. These can overlap in size and protein composition, thus rendering their specific analysis very difficult (59). In order to clearly establish the exosomal association of APE1 within the bulky vesicular population, we analyzed the physical characteristics of the EVs (*e.g.*, size and/or density ranges) and positivity to protein markers, as indicated (60). Through combined biochemical and different technological approaches (flow cytometry and electron microscopy), we assessed the specific expression of APE1 in small EVs presenting density (1.08–1.18 mg/ml) and dimension (120 nm) typical of exosomes.

The biological function exerted by APE1 through exosomes remains to be elucidated. A recent study suggested a role of sAPE1 in HCC as a paracrine proinflammatory molecule, modulating the inflammatory status in cancer microenvironment (29) and in human monocytes (26). Several studies reported a role for secreted APE1 in inducing an apoptotic signaling in triple-negative breast cancer (TNBC) cells through RAGE receptor binding (61), whereas same interaction observed in monocytic cells triggered signaling cascade involved in inflammatory response (26). We can hypothesize similar biological functions for exosome-associated APE1. In addition, our finding on the vesicular-enclosed topology of APE1 suggests the existence of a novel RAGE-independent mechanism, which deserves further studies actually ongoing in our laboratory. The extracellular release of APE1, not only through exosomes, but also as soluble protein, cannot be excluded, at present. Moreover, it was observed that APE1 extracellular release could be mediated by acetylation occurring on the N-terminal region of the protein (61), but no clear evidence about either the extracellular APE1 biological function or the mechanism responsible for its release has been elucidated, yet. Other evidence claims that extracellular APE1 can control TNF-α-induced inflammatory response, modulating the activation status of TNFR receptor through its redox activity (27). Additional data suggest that exogenous APE1 can participate in the early phases of the inflammation process, by inducing the activation of IL-8 and IL-6 gene expression through NF-κB (27). All these observations, together with our findings about the effects of genotoxic treatments on APE1 release, could suggest that APE1 may act as a novel unsuspected damage associated molecular pathway (DAMP) factor. This may indicate a novel noncanonical APE1 function as a paracrine molecule, possibly involved in the inflammatory response in tumor microenvironment (62) and in tumor growth.

The sorting of APE1 to exosomes opens a new perspective on the molecular mechanisms involved in APE1 secretion, unknown so far. The APE1 protein lacks the classical secretory signal even if its secretion was already described in several works (25, 31). Although the route followed by APE1 in vesicles association is unknown, we can speculate that APE1 could follow the exosome packaging regulated through various signaling networks. In particular, the biogenesis of exosomes does occur within endosomes, later developing toward MVBs, whose maturation is mainly driven by an ESCRT-dependent mechanism. In support of this, ESCRT proteins, which are crucial for packing MVBs, were found within exosomes (63). However, several studies reported evidence for an ESCRT-independent biogenesis (64, 65) and a role for the sphingolipid ceramide, highly enriched in exosome membranes (66). This sphingolipid together with high cholesterol level and other sphingolipids forms lipid rafts inside endosomal membranes, which force the coalescence of small into larger microdomains, ultimately inducing budding and formation of ILVs, later secreted as exosomes. Additional biochemical characterization, actually ongoing in our lab, will clarify the route followed by APE1 in vesicles association and secretion.

Regarding the mechanism responsible for APE1 sorting into the exosomes, we found that the APE1-exosome secretion process is a general mechanism in mammalian cells and that APE1 can be found either as single p37 protein form or as p33/p37 forms within exosomes due, at least in part, to proteosomal activity. However, at present, we cannot exclude that proteolytic events acting on APE1-N-terminal region could preferentially occur at intracellular level or within EVs upon secretion. Recently, some works highlighted that EVs could contain different kind of proteases, including component of the proteasome (48, 49), some of which were exposed to the EVs membrane and contributing to the remodeling of extracellular matrix promoting tumor invasiveness and sustaining inflammation processes (48, 49). We observed that APE1 can undergo proteolytic cleavage, within cells (this paper and (12)) and exosomes and that this activity increased under genotoxic stress conditions (not shown), but additional work is needed along these lines to clarify the relevance of this phenomenon regulating APE1 sorting and its function within exosomes. Moreover, a possible relevance of additional posttranslational modifications in regulating these phenomena cannot be excluded, at present. The enhanced release of exosome-APE1 p33 form upon different genotoxic stresses, specifically Doxo and CDDP treatments used in the present study, is in line with a previous study showing an increased release of exosomes of nuclear origin deriving from micronuclei instability after genotoxic damage in cancer cells (67). It is conceivable that these vesicles might be highly shuttled between cells within tumor mass and deliver their content within target cells. This process may fulfill the cancer cells requirement of high amount of APE1 to counteract the DNA damage inferred by drugs in a paracrine manner, suggesting that APE1 secretion could represent a novel DAMP mechanism, which deserves further in-depth study. Importantly, these data provide the rationale to investigate APE1 p33 accumulation in exosomes from a liquid biopsy to monitor patients subjected to genotoxic drugs.

In conclusion, we here demonstrated that APE1 is highly secreted through exosomes from cancer cells after genotoxic stress in different functional forms and that it is endowed with enzymatic activity, possibly representing a novel DAMP factor. These data add new light in the complex biological functions of APE1 that could play important roles in chemoresistance processes and tumor development.

## Experimental procedures

### Cell culture and treatments

JHH-6, an undifferentiated HCC cell line, was grown in William’s medium E (Sigma-Aldrich, Milan, Italy), supplemented with 10% FBS, 2 mM L-glutamine, 100 U/ml penicillin, 100 μg/ml streptomycin (Euroclone, Milan, Italy). A549, human epithelial lung cell line and mouse embryonic fibroblast (MEF) expressing (MEF NPM1+/+) or not NPM1 (MEF NPM1−/−) (43) were grown in RPMI 1640 supplemented with 10% FBS, 2 mM L-glutamine, 100 U/ml penicillin, 100 μg/ml streptomycin (Euroclone). HCT116 ^p53+/+^ and HCT116 ^p53−/−^, human epithelial colon cancer cell lines, were grown in Dulbecco’s Modified medium (DMEM) supplemented with 10% FBS, 2 mM L-glutamine, 100 U/ml penicillin, 100 μg/ml streptomycin (Euroclone). Acute myeloid leukemia cell lines OCI-AML2, and OCI-AML3, the last one characterized by having a mutation on the third helix of the C-terminal domain (h3 mutA and H3 mutE) of NPM1 protein (36, 68), were grown in α-MEM with ribonucleosides, 20% FBS, 2 mM L-glutamine, 100 U/ml penicillin, 100 μg/ml streptomycin (Euroclone). CH12F3^Δ/+/+^, murine B cell line, biallelic for *ape1* gene, and CH12F3^Δ/Δ/Δ^ murine B cell line, allelic null for *ape1*, were grown in RPMI 1640 medium supplemented with 10% FBS, nonessential amino acids (Thermo Fisher Scientific, Waltham, MA, United States), 1 mM sodium pyruvate, 50 μM β-mercaptoethanol, 25 mM HEPES, 2 mM L-glutamine, 100 U/ml penicillin, 100 μg/ml streptomycin (Euroclone). HeLa, human adenocarcinoma cells, were previously engineered for APE1-Dendra2 and Dendra2 (41), and were grown in DMEM high glucose (DMEM) supplemented with 10% FBS, 2 mM L-glutamine, 100 U/ml penicillin, 100 μg/ml streptomycin (Euroclone). Cells were free from *mycoplasma* contamination. The test was carried out using N-GARDE *Mycoplasma* PCR Reagent (Euroclone).

### Viability assay

JHH-6 or A549 was seeded at the density of 4∗10^3^ cells in 96-well plates. The day after seeding, cells were treated for 24, 48, and 72 h with the following genotoxic agents: Doxorubicin hydrochloride (Sigma Aldrich), dissolved in UltraPure DNase/RNase-Free Distilled Water (Invitrogen, Thermo Fisher Scientific) or with cis-Diammineplatinum (II) dichloride (CDDP) (Sigma Aldrich), dissolved in dimethylformamide (DMF), at the indicated concentrations. The cellular viability was assayed by using CellTiter 96 AQueous One Solution Cell Proliferation Assay (Promega, Milan, Italy). Absorbance at 490 nm, indicative of cellular metabolic activity, was measured with EnSpire 2300 Multilabel reader, (PerkinElmer, Waltham, MA, United States). Each treatment was performed in two biological replicates, and for each experiment a technical quadruplicate was performed. Each absorbance value registered was standardized with the absorbance value obtained from wells containing only medium.

### Exosomes isolation

Exosomes were isolated from medium supplemented with 10% FBS exosome-depleted (Thermo Fisher Scientific), 100 U/ml penicillin, 100 μg/ml streptomycin (Euroclone), using Total Exosome Isolation kit (Thermo Fisher Scientific), according to the manufacturer’s instructions. Polydisperse EVs in the range of 80–800 nm were obtained by NBI (38). Briefly, nickel-charged beads were added to the media to capture EVs during 30 min incubation at room temperature in shaking, then EVs from recovered beads were eluted using a pH 7.4 elution buffer containing EDTA and sodium citrate as chelating agents.

Exosomes were also obtained by ultracentrifugation-based method (40). Briefly, cell culture conditioned medium (24–48 h) was subjected to serial centrifugation steps at 4 °C. The first one was at 700 x *g* for 10 min. The collected supernatant was subjected to 2,000 x g for 20 min to remove protein aggregates, and super was syringe-filtered (0.2 micron). Samples were then centrifuged at 15,000 x *g* for 20 min. Smaller vesicles containing exosomes were isolated by high-speed ultracentrifugation (100,000 x *g* for 3 h). Pellet was washed with PBS without resuspension at 100,000 x g for 3 h.

### Optiprep gradient centrifugation

We carried out a discontinuous iodixanol gradient floatation as described in (69). Briefly, solutions of 40, 30, 10% iodixanol were made by mixing appropriate amounts of a homogenization buffer (0.25 M sucrose, 1 mM EDTA, 10 mM Tris-HCl pH 7.4) and an iodixanol working solution (0.25 M sucrose, 6 mM EDTA, 60 mM Tris pH 7.4 plus stock solution of Optiprep 60% (w/v), (Axis-Shield PoC, Norway). Exosomes isolated with the ultracentrifugation-based method were resuspended in PBS (260 μl) and added to 1 ml of 60% Optiprep and placed at the bottom of a polyallomer tube. The gradient was formed by layering 0.5 ml of 40%, 0.5 ml of 30%, 1.8 ml of 10% solutions on top of each other and centrifuged for 17 h at 192,000 × *g* in a SW60 rotor (Beckman Coulter, Cassina de’ Pecchi, MI, Italy). Thirteen fractions (330 μl) were collected from the top of the tube. The refractive index of each fraction was assessed with a refractometer (Carl Zeiss, Oberkochen, Germany), and the relative density was calculated using the linear relationship between the refractive index and the density (70).

### Exosome quantification and characterization

Exosomes range size analysis and quantification were assayed by performing the nanoparticle tracking analysis (NTA) with the Nano-Sight instrument, version NTA 3.2 Dev Build 3.2.16, and for samples obtained by ultracentrifugation with the Nano-Sight NS300 (Malvern, Worcestershire, UK). EVs isolated by NBI were characterized by TRPS technology, using qNANO instrument (IZON Science, Oxford, United Kingdom) and NP250 nanopores. Stretch conditions and quality of acquisitions were assessed based on CPC200 calibration beads (IZON Science).

### Amplified luminescent proximity homogeneous assay (alpha)

Glutathione-Donor beads (PerkinElmer, #6765300) and Protein G-Acceptor beads (PerkinElmer, #AL102 C) were used in saturation binding experiments to detect and quantify the secreted, recombinant APE1 protein. Briefly, a mix of Donor and Acceptor beads, at final concentration of 10 mg/ml each, was added to 3 nM of APE1 antibody (13B8E5C2, Novus, Colorado, USA) preincubated with increasing concentration of rGST-APE1^WT^ or GST to reach hooking point (38). In case of EV experiments, aliquots of vesicles were denatured or not at 90 °C for 5 min and equilibrated at RT before adding the alpha mix of beads plus antibody (40). All the reactions were performed in 20 μl as final volume in 384-Optiplates (PerkinElmer) and incubated for 1 h at RT before acquiring the results with an Enspire Plate Reader (PerkinElmer). Curves were analyzed by GraphPad Prism software v.5.

### Whole-cell extracts preparation (WCE)

Cells were harvested, centrifuged at 1,200 rpm for 3 min, washed with PBS, and resuspended with lysis buffer (50 mM Tris HCl pH 7.5, 150 mM NaCl, 1 mM EDTA pH 8.0, 1% Triton X-100) supplemented with 1 mM protease inhibitor cocktail (Sigma), 0.5 mM phenylmethylsulfonyl fluoride (PMSF). After 20 min of incubation on ice, the lysate was centrifuged at 13,000 rpm to remove debris, and the whole-cell extract (WCE) was collected and kept at –80 °C.

### Exosome extracts preparation (EXE)

Once the EVs were obtained, laemmli sample buffer (2% SDS, 10% Glycerol, 0.5 M Tris-HCl pH 6.8, 3.5% β-mercaptoethanol, bromophenol blue) was added to shatter the vesicles and to denature their protein content. The samples were later boiled for 5 min at 95 °C and kept on ice until use.

### Western blot analysis

Protein extracts were suitably quantified with Bradford solution (Biorad, California, USA), according to the manufacturer’s instructions, loaded in 10% gel (SureCast Acrylamide Solution (40%), Invitrogen), and transferred to nitrocellulose membrane (Amersham, Little Chalfont, UK). Western blot analyses were executed using the listed antibodies: anti-APE1 (13B8E5C2, Novus), anti-APE1 (N-terminus aa 1–14) (NB100–897, Novus), anti-β-Tubulin (T0198, Sigma-Aldrich), anti-Alix (1A12) (SC-53540, Santa Cruz, Texas, USA), anti-GM130 (B-10) (SC-55591, Santa Cruz), anti-Syntenin (EPR8102) (ab133267, Abcam), and anti-GST (ab19256, Abcam). After incubation with primary antibodies, membranes were washed three times with PBS 0.1% Tween-20, (Sigma Aldrich), incubated for 1 h at room temperature with the appropriate IRDye800/IRDye600 labeled secondary antibodies (diluted to 1:2000). The acquisition of the images and the quantifications analyses were achieved using Odyssey CLx Infrared Imaging system (LI-COR GmbH, Germany).

### FACS analysis of APE1-Dendra2

HeLa cell lines stably transfected with APE1-Dendra2 and Dendra2 constructs (41) were cultured for 48 h with medium supplemented with 10% FBS, exosome-depleted (Thermo Fisher Scientific). Exosomes were obtained with ultracentrifugation method. The presence of fluorescent events in exosomal pellet was assessed by flow cytometry analysis as reported (71) with minor modifications. Isolated exosomes were resuspended with PBS (200 μl), then analyzed and counted with CytoFLEX LX flow cytometer (Beckman Coulter). The analysis was performed by plotting fluorescence at 525/540 nm (FL1) versus log scale side scatter to determine the fluorescence threshold value able to exclude the background noise. The instrument, calibrated to delivery sample for absolute cell counts without using beads, was set at stopping gate of 60 μl and fluorescence events in 595/540 nm channel were recorded. To ensure the instrument resolution and reliability, commercially available size standard fluorescent beads (green fluorescent 505/515 flow cytometry submicron particle size reference kit (Life Technologies)) were used.

### Electron microscopy

Exosomes isolated by ultracentrifugation were analyzed by scanning electron microscopy (SEM). Briefly, exosomes were left to adhere on polylysine-treated round glass coverslips (Ø10 mm) at room temperature. Then samples were fixed with 2.5% glutaraldehyde in 0.1 M Na-cacodylate buffer for 30 min and post-fixed with 1% OsO_4_ in 0.1 M sodium cacodylate buffer for 1 h. After fixation, samples were dehydrated through a graded series of ethanol solutions (from 30 % to 100%). After dehydratation, absolute ethanol was gradually substituted by a 1:1 solution of hexamethyldisilazane (HMDS)/absolute ethanol and then by pure HMDS (72). Dried samples were mounted on stubs, coated with 10 nm gold, and analyzed in a GeminiSEM 450 (Carl Zeiss).

### Coomassie brilliant blue staining

Coomassie staining (0.05% Coomassie brilliant blue, 5% acetic acid, 50% methanol) was carried out for 15 min, then the 10% polyacrylamide gels were incubated for 30 min in destaining solution (25% methanol, 7% acetic acid). The acquisition of the image was achieved using Odyssey CLx Infrared Imaging system (LI-COR GmbH, Germany).

### Recombinant proteins

Expression of human recombinant APE1 (rAPE1) was produced as explained in (73, 74, 75).

*E. coli* RNase HII and bovine pancreas RNase A were purchased from New England BioLabs Inc (Milan, Italy) and from Sigma (Milan, Italy), respectively.

### Proteolytic activity in exosomes

Extracellular vesicles were disrupted with 0.1% of PBS-Triton X-100 (Sigma Aldrich) and their protein content was later quantified with Bradford solution (Biorad). Different concentrations of EVs proteins (0.1, 0.5, 1 μg) were incubated with 200 ng of rGST-APE1^WT^, rGST-APE1^K4pleA^ proteins, and the reactions were performed at 37 °C for 4 h. The reactions were stopped adding Laemmli sample buffer. Samples were later boiled for 5 min at 95 °C. Each sample was loaded into 10% polyacrylamide gel, SDS PAGE was carried out, and finally western blot was executed.

### Enzymatic activity assays

To measure enzymatic activity of EXE on different modified DNA substrates (25 nM), each reaction was prepared following different concentration of the extract, expressed in ng, as specified in the legend of each panel. Reactions were performed in a buffer containing 20 mM Tris-HCl pH 7.4, 25 mM KCl, 4 mM MgCl_2_, 0.1% BSA, 0.01% Tween20, for the indicated time at 37 °C. To measure enzymatic activity of EXE on modified RNA substrate (250 nM), each reaction was prepared following different concentrations of the extract in a buffer containing 10 mM Tris-HCl pH 7.4, 25 mM KCl, 1 mM MgCl_2_, for the indicated time at 37 °C. Final volume for each reaction was 10 μl. At the end of all reactions, samples were blocked with a stop solution, containing 99.5% v/v Formamide (Sigma-Aldrich, Milan, Italy), supplemented with 10X Orange Loading Dye (LI-COR Biosciences, Milan, Italy) and heated at 95 °C for 5 min. Then, all samples were loaded onto a 7 M denaturing 20% polyacrylamide gel in TBE buffer pH 8.0 and run at 4 °C at 300V for 1 h. Then, the non-incised substrate (S) and the incision product (P) bands were quantified using Image Studio software (LI-COR GmbH, Germany).

### Statistical analysis

Statistical analyses were performed by using the Student’s *t*-test in GraphPad Prism software. When *p* < 0.05, data were considered as statistically significant.

## Data availability statement

All data are contained within the article and in supporting information file.

## Supporting information

This article contains supporting information.

## Conflict of interest

The authors declare that no conflict of interest exists, except for V. G. D. for a patent application (P019950W0) on NBI method.
