# Supplementary material for: Enzymatically active apurinic/apyrimidinic endodeoxyribonuclease 1 is released by mammalian cells through exosomes
Source: J Biol Chem. 2021 Mar 19;296:100569. doi: 10.1016/j.jbc.2021.100569 (PMC8080531; doi:10.1016/j.jbc.2021.100569)
Supplement: Figures S1–S12 [file mmc1.pdf]

Enzymatically active apurinic/apyrimidinic endodeoxyribonuclease 1 is released by mammalian cells through exosomes

Giovanna Mangiapane[1], Isabella Parolini[2], Kristel Conte[1], Matilde Clarissa Malfatti[1], Jessica Corsi[3], Massimo Sanchez[4], Agostina Pietrantoni[4], Vito G. D'Agostino[3] and Gianluca Tell[1]

[1] Laboratory of Molecular Biology and DNA repair, Department of Medicine (DAME), University of Udine, Udine, Italy.

[2] Department of Oncology and Molecular Medicine, Istituto Superiore di Sanità, Rome, Italy

[3] Department of Cellular, Computational and Integrative Biology (CIBIO), University of Trento, Trento, Italy.

[4] Core Facilities, Istituto Superiore di Sanità, Rome, Italy.

Running Title: APE1 endonuclease is secreted through exosomes

Keywords: Apurinic/apyrimidinic endodeoxyribonuclease 1, Base excision repair biomarker, extracellular vesicles, exosomes, genotoxic damage, proteasome

To whom correspondence should be addressed: Prof. Gianluca Tell, Head of the Laboratory of Molecular Biology and DNA repair, Dept. of Medicine, University of Udine, Piazzale M. Kolbe 4, 33100 Udine – Italy; gianluca.tell@uniud.it: Tel.+39 0432 494311; Fax. +39 0432 494301

Supporting information

Supplementary Figure

S1

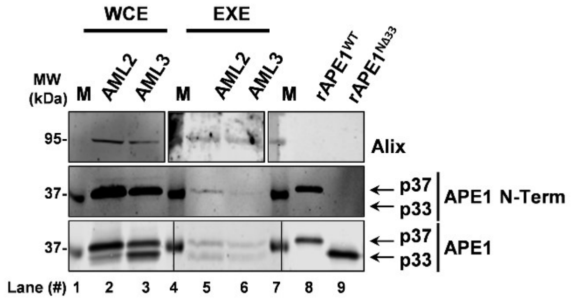

Supplementary figure S1: Western blotting analyses was carried out in WCE and EXE of AML2 and AML3 cell lines to characterize the APE1 p33 form. The NB100-897 (Novus) antibody, recognizing an epitope occurring into the N-terminal region of the protein, was used to detect only the p37 full length form of the protein. The I3B8E5C2 (Novus) APE1 monoclonal antibody which recognizes an epitope within amino acids 80-100 of the protein, was used to detect indiscriminately both p33 and p37 forms. rAPE1^{WT} and rAPE1$^{N\Delta 33}$ were used respectively as a positive control for APE1 and APE1 N-term antibodies. Alix detection was performed as loading control. The incubations with I3B8E5C2 APE1 monoclonal and Alix antibodies were carried out after cutting the membrane in correspondence of the markers (vertical black lines indicate the cutting sites) to avoid antibody seizures from WCE and recombinant protein samples respect to less APE1 concentrated EXE samples.

S2

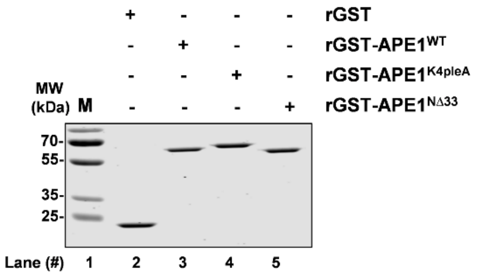

Supplementary figure S2: Coomassie staining for rGST-APE1 proteins was carried out loading 1.5 µg of rGST, rGST-APE1^{WT}, rGST-APE1^{k4pleA}, rGST-APE1$^{N\Delta33}$ proteins into a 10% polyacrylamide gel.

S3

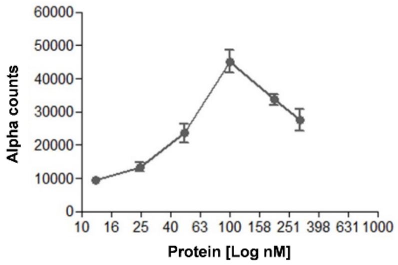

Supplementary figure S3: Calculation of the hook point in the AlphaScreen assay. Glutathione-Donor beads and Protein G-Acceptor beads were used in saturation binding experiments to detect recombinant APE1 protein. Donor and Acceptor beads, at final concentration of 10 mg/ml each, were added to 3 nM of APE1 antibody pre-incubated with increasing concentration of rGST-APE1^{WT} or rGST to reach optimal conditions for detecting interacting partners.

S4

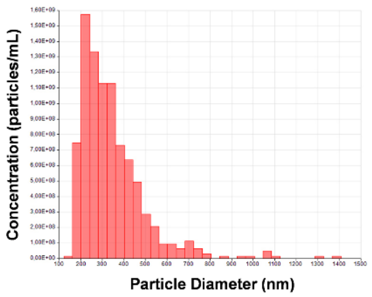

Supplementary figure S4: Tunable Resistive Pulse Sensing (TRPS) measurement of NBI-isolated EVs using qNANO instrument (iZON Science). The range of size shown by the NP250 nanopore was calibrated with CPC200 beads (iZON Science) under constant stretch condition and noise, maintaining linear acquisition rates of particles' acquisition.

S5

	APE1	
	**p33**	**p37**
**AML2**	45.02 ± 6.08	54.98 ± 6.08
**AML3**	49.91 ± 2.52	50.09 ± 2.52

Supplementary figure S5: Chart showing data obtained by densitometry analysis performed for the quantification of APE1 p33 and p37 signals relative to AML2 and AML3 EXE. Data are expressed in percentage as mean ± SD of three independent technical replicas.

S6

JHH-6	APE1	
	**p33**	**p37**
**CTR**	69.63 ± 20.96	30.37 ± 20.96
**MG-132**	36.12 ± 15.58	63.88 ± 15.58

Supplementary figure S6: Chart showing data obtained by densitometry analysis performed for the quantification of APE1 p33 and p37 signals relative to EXE derived from JHH-6 untreated control (CTR) and MG-132 treated cells. Data are expressed in percentage as mean ± SD of three independent replicas.

S7

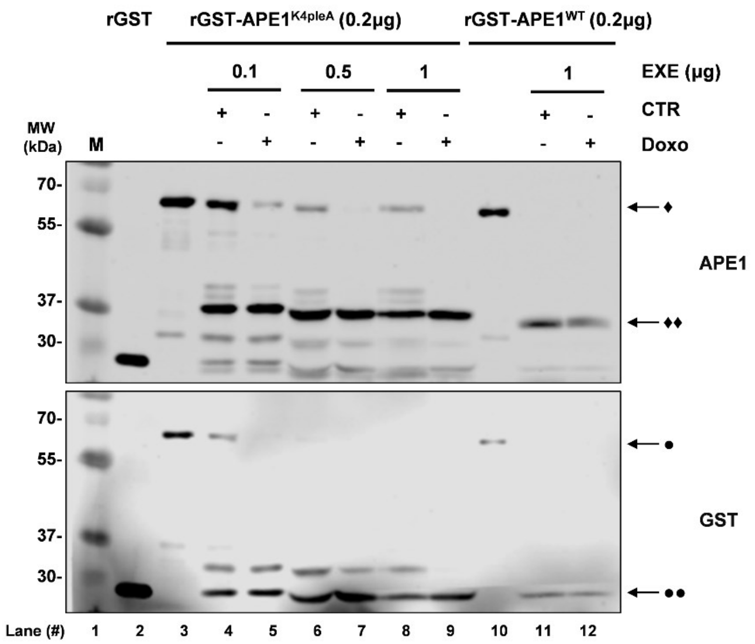

Supplementary figure S7: *In vitro* proteolytic activity upon rGST-APE1^{WT} (0.2 µg) (apparent molecular weight 65 kDa) and rGST-APE1^{K4pleA} (0.2 µg) (apparent molecular weight 69 kDa) exerted by the indicated quantity of EXE expressed in µg, derived from Doxo treated and untreated cells (CTR). The reactions were performed at 37°C for 4 hr. The substrates and the products of reactions have been detected by Western blotting using APE1 and GST antibodies and are respectively indicate as: (♦), (♦♦) for APE1 and (●), (●●) for GST.

S8

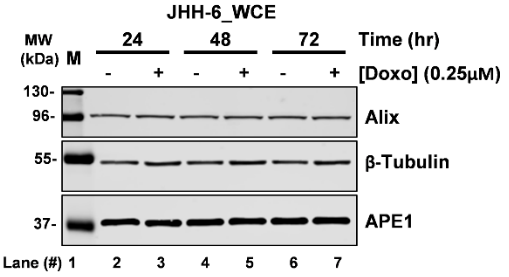

Supplementary figure S8: Analysis of APE1 accumulation and study of p37 and p33 distributions in JHH-6 WCE after treatments with sub-lethal concentration of Doxo at the indicated time points. APE1 p37 and APE1 p33 were analyzed by western blotting. Alix and β-Tubulin were detected as loading controls.

S9

JHH-6		APE1	
		p33	p37
24h	CTR	2.47 ± 3.35	97.53 ± 3.35
	Doxo	81.75 ± 2.34	18.25 ± 2.34
48h	CTR	3.68 ± 1.58	96.32 ± 1.58
	Doxo	86.83 ± 3.10	13.17 ± 3.10
72h	CTR	5.70 ± 1.76	94.30 ± 1.76
	Doxo	12.98 ± 3.54	87.02 ± 3.54

Supplementary figure S9: Chart showing data obtained by densitometry analysis performed for the quantification of APE1 p33 and p37 signals relative to EXE derived from JHH-6 untreated control (CTR) and Doxo treated cells at the indicated time points. Data are expressed in percentage as mean ± SD of three independent replicas.

S10

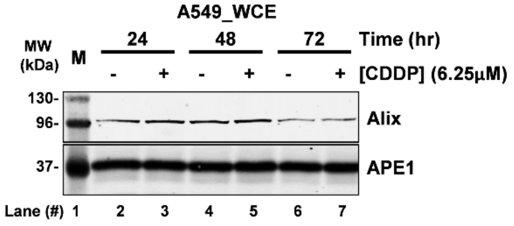

Supplementary figure S10: Analysis of APE1 accumulation and study of p37 and p33 distributions in A549 WCE after treatments with sub-lethal concentration of CDDP at the indicated time points. APE1 p37 and APE1 p33 were analyzed by western blotting. Alix was detected as loading control.

S11

A549		APE1	
		p33	p37
24h	CTR	32.45 ±7.74	67.55 ± 7.74
	CDDP	50.87 ± 28.45	49.13 ± 28.45
48h	CTR	39.33 ± 7.48	60.67 ± 7.48
	CDDP	97.40 ± 0.97	2.60 ± 0.97
72h	CTR	44.92 ± 14.84	55.08 ± 14.84
	CDDP	70.30 ± 37.94	29.70 ± 37.94

Supplementary figure S11: Chart showing data obtained by densitometry analysis performed for the quantification of APE1 p33 and p37 signals relative to EXE derived from A549 untreated control (CTR) and CDDP treated cells at the indicated time points. Data are expressed in percentage as mean ± SD of three independent technical replicas.

S12

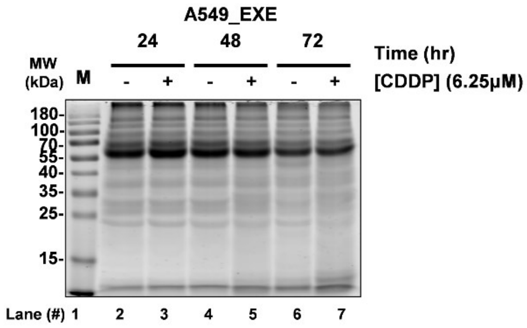

Supplementary figure S12: Coomassie staining for A549 EXE derived from untreated control and CDDP treated cells at the indicated time points was carried out loading 15 µg of EXE samples into a 12% polyacrylamide gel.
